# Four Thousand Years of Concepts Relating to Rabies in Animals and Humans, Its Prevention and Its Cure

**DOI:** 10.3390/tropicalmed2020005

**Published:** 2017-03-24

**Authors:** Arnaud Tarantola

**Affiliations:** 1 Epidemiology & Public Health Unit, Institut Pasteur du Cambodge, BP983 Phnom Penh, Cambodia; atarantola@pasteur-kh.org or atarantola@pasteur.nc; Tel.: +687-50-78-88; 2 Unité de Recherche et d’Expertise en Maladies Infectieuses (UREMI), Institut Pasteur de Nouvelle-Calédonie, 9800 Nouméa, New Caledonia

**Keywords:** rabies, vaccine, history, One Health, post-exposure prophylaxis, Galtier, Roux, Pasteur, Semple, dog

## Abstract

The epitome of the One Health paradigm—and of its shortcomings—rabies has been known to humankind for at least 4000 years. We review the evolution through history of concepts leading to our current understanding of rabies in dogs and humans and its prevention, as transmitted by accessible and surviving written texts. The tools and concepts currently available to control rabies were developed at the end of the 19th Century, including the first live, attenuated vaccine ever developed for humans and the first post-exposure prophylaxis (PEP) regimen. No progress, however, has been made in etiological treatment, leaving clinicians who provide care to animals or patients with symptomatic rabies as powerless today as their colleagues in Mesopotamia, 40 centuries ago. Rabies remains to date the most lethal infectious disease known to humans. Widespread access to timely, effective, and affordable PEP in rural areas of developing countries is urgently needed.

## *Preamble* 

*Rabies is an ancient and much-feared disease. Over the centuries, many different authors—clinicians, veterinarians, surgeons, pharmacists but also writers, philosophers, and poets—have mentioned rabies in their writings. The sequence of research and surviving writings on rabies described below is far from exhaustive. Rather, it aims to identify the work of those who made remarkable contributions to the current stage of knowledge on dog-mediated rabies, its cause and its prevention, control or management. Sources that conducted research on rabies but proposed alternate views of causation now considered misleading (such as spontaneous generation) have purposefully been left aside. Furthermore, no further potential sources from Ancient China, India, pre-Columbian America, or Africa could be identified or accessed*.

## 1. Rabies in Sumerian and Akkadian Civilizations

**Figure 1:**
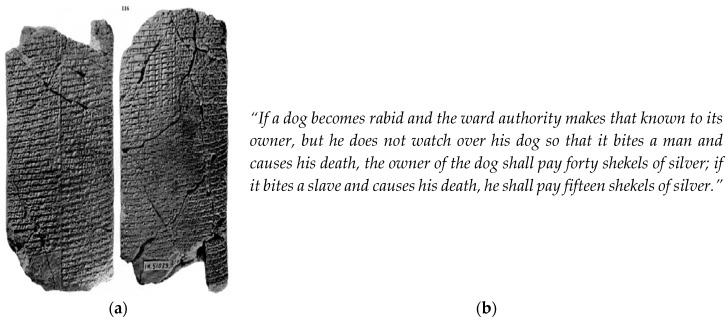
Excerpts from the Sumerian Laws of Eshnunna, Northern Babylonia ca. 1930 BCE. (**a**) Tablets of the Laws of Eshnunna; (**b**) One possible translation of Paragraphs 56–57 of the Laws of Eshnunna (A iv 20–24) [10]. Another possible translation speaks of a dog becoming “furious” or “vicious” [8,9]. Even 15 shekels was a considerable sum: The Hammurabi code mentions the cost of a boat of sixty “gur” at two shekels. (Source: http://legacy.fordham.edu/halsall/ancient/hamcode.asp). Acknowledgement: Dr. Mark Weeden, Lecturer in Ancient Near Eastern Studies, School of Oriental and African Studies, London, UK.

**Figure 2:**
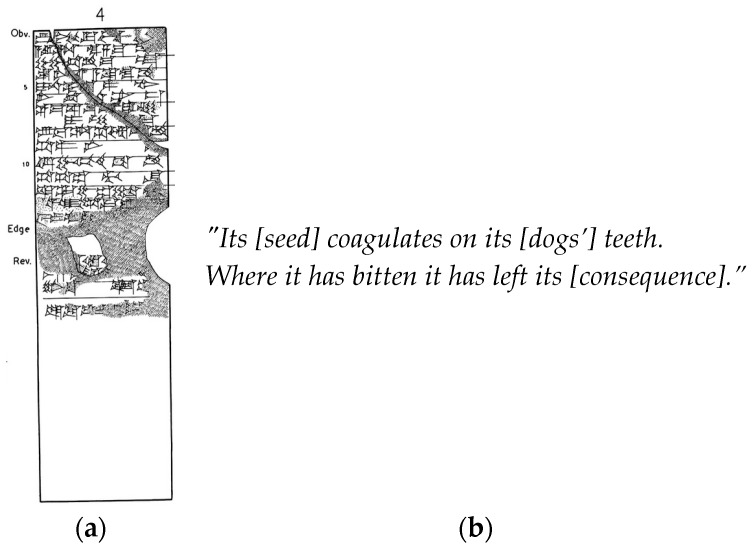
“Dog incantation”, ca. 1900–1600 BCE (**a**) Tablet; (**b**) Translation, adapted from [11].

Humans have lived alongside domesticated dogs for 14,000 years at least, with estimates reaching back to 32,000 years [1,2]. They have also long been familiar with their diseases, which became more prevalent as populations and their animals congregated in the cities that arose in Mesopotamia [3,4,5,6]. Two cuneiform tablets (Figure 1) discovered at Tell Abū Harmal, Baghdad Governorate, Iraq in 1945 and 1947, recount the Laws of Eshnunna, a Sumerian and later Akkadian city-state located in present Tell Asmar, Iraq [7]. This city was most prominent during the Isin-Larsa period, ca. 1950–1850 BCE and the tablet is dated ca. 1770 BCE [8]. Distinct copies of another source date back to ca. 1930 BCE. These describe Sumerian rules and regulations attesting to the fact that a causal link between the bite of a rabid animal and a human death from rabies was well recognized almost 4000 years ago [9]:

At least five old Mesopotamian “dog incantations” (ca. 1900–1600 BCE) such as the one below (Figure 2) clearly reflect the notion of rabies being caused by something present in the saliva of the afflicted animal, akin to the poison transmitted by a snakebite or scorpion sting [9,10,11,12]. An herb seems to have been used after a dog bite and the biting dog’s movement was restricted [12]. Dogs were thought more likely to become rabid when a lunar eclipse occurred at year’s end [9].

**Figure 3:**
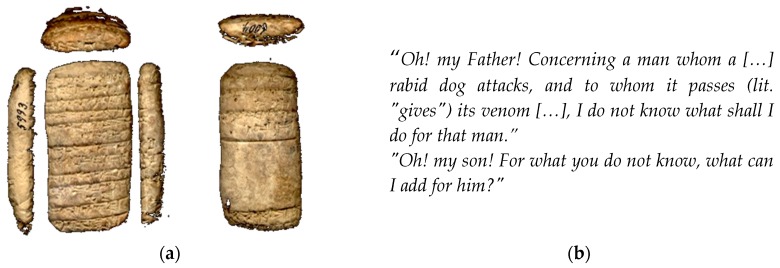
Ur incantations. (**a**) Tablets of the Ur III incantations (http://cdli.ucla.edu/P142047); (**b**) Translation. Acknowledgement: Prof. N. Veldhuis, Professor of Assyriology, University of California, Berkeley, CA, USA.

Finally, clay tablets (Figure 3) unearthed by H.V. Hilprecht in 1889 at the Nippur site (3rd dynasty of Ur III, 21st- 20th-century BCE) of what is now Nuffar in Iraq display Akkadian incantations, to which healers resorted when medicine failed [9,13,14]. This dialogue between Marduk, the God of Healing, and his father Enki was recited by priests over (thus blessed) water which was then administered orally. These incantations are striking, marked as they are by the caveat of likely—however divine—failure, and certain death should rabies develop.

**Figure 4:**
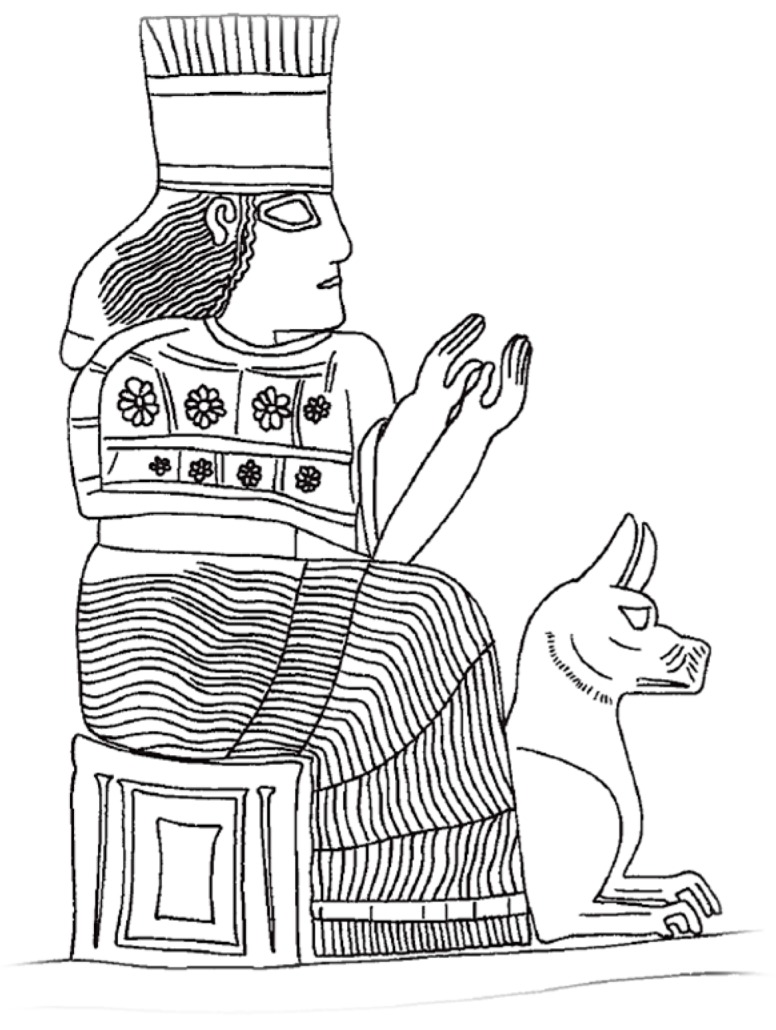
Goddess Gula represented on her throne, a dog at her feet on a *kudurru* of Nebuchadnezzar I (12th Century, BCE) [16]. Acknowledgement: Prof. Tallay Ornan, Hebrew University of Jerusalem, Department of Archaeology & the Ancient Near East Department.

Just like Yama, a Hindu god of death, the Babylonian Goddess Gula, patroness of doctors and a healing deity, was represented in the 14th–7thC BCE with a dog at her feet [15,16] (Figure 4). In one ancient tale, a Nippur man bitten by a dog, self-referred for treatment to a temple in Isin, the city of Gula [9]. As ancient deities of the Near East were shown mounting or otherwise dominating animals to demonstrate their power, it can be hypothesized that this association represented dogs both positively (the dog as a protector) and negatively (the dog as a source of danger, including rabies) (Prof. T. Ornan, personal communication, 11 December 2015).

Although this remains disputed [17], the origin of “rabias”, the Latin word for rabies, may originate from “rabhas” or “rabhasa” (रभस) in Sanskrit (http://www.webcitation.org/6os2XRrN8), perhaps crossing Indo-European cultures and centuries [18]. Indeed, rabies is mentioned in many ancient texts, from the Vedic period (in ancient India ca. 1750–500 BCE) [19,20,21], to ancient China [22,23,24,25], Egypt [26] and the Middle East [27] as well as Greece and Rome [19,28]. Attempts at prevention or treatment of clinical rabies, however, remained faith-based, magical or otherwise exotic [19,29,30,31].

## 2. Rabies in Classical Antiquity

Aristotle, Hippocrates, Pliny, Ovid, Cicero... a great many texts by physicians and other authors of classical antiquity attest to a progressively improved comprehension of rabies. They—and especially Caelius Aurelianus, who also wrote an early description of palliative care in rabies patients [32]–provide accurate and detailed descriptions of symptoms, whether in dogs or in humans [19,28,31,33,34]. Galen noted the absence of symptoms in bite victims before the onset of rabies [34]. Both Dioscorides (ca. 4–90 CE) and Philomenos (1stC CE) discuss a latency period of varying duration after an infective bite, generally lasting six weeks but sometimes lasting up to several years [32]. In his “Emergency Formulas to Keep up One’s Sleeves” (Zhŏu Hòu Jiù Zú Fān, 肘後備急方), Ge Hong (葛洪) of the Jin Dynasty (around 300 CE) also described prolonged incubation periods in humans (but unfortunately recommended the application of the biting dog’s brain tissue to the bite wound to prevent rabies) [35].

Primary prevention of rabies through the prevention of bites by suspected rabid dogs was recommended in the Persian Avesta, composed in 200–400 CE, perhaps from much more ancient texts [36]. Around 60 CE, Columella’s *De Re Rustica* described shepherds’ habit of cutting puppies’ tails when they are 40 days old, as a preventive measure against rabies in dogs should they be bitten, perhaps one of the earliest known example of One Health, or at least One Medicine, which sees disease prevention in humans as intimately linked with the health of the animals to which they are exposed [32,37,38,39]. Many different treatments were on offer to prevent rabies in dogs after they had been bitten [40].

Rabies prevention after a bite in humans made few advances. Similar incantations to that found at Nippur / Nuffar were spoken in Greek-speaking Egypt around the 3rdC CE [41]. Along with Aulus Cornelius Celsus in his *De Medicina* (published between 18 and 39 CE in Rome) [42,43], the only author who may have had some impact on the replication of viral inocula in wounds made by rabid animals, was Pedianus Dioscorides (ca. 40–90 A.D.), of Anazarba in Cilicia, founded by the Assyrians but a then Roman city, now in Adana Province of southern Turkey. A physician and a pharmacologist, he is said to have described rabies accurately and like Celsus, proposed cauterization of the bitten part as prevention [28]. But all attempts at treatment of clinically-declared rabies cases remained based on hopeful conjecture [44] or were denounced as unnecessarily brutal, as by Asclepiades of Bithynia in the mid-2ndC CE [45].

## 3. The Middle Ages

**Figure 5:**
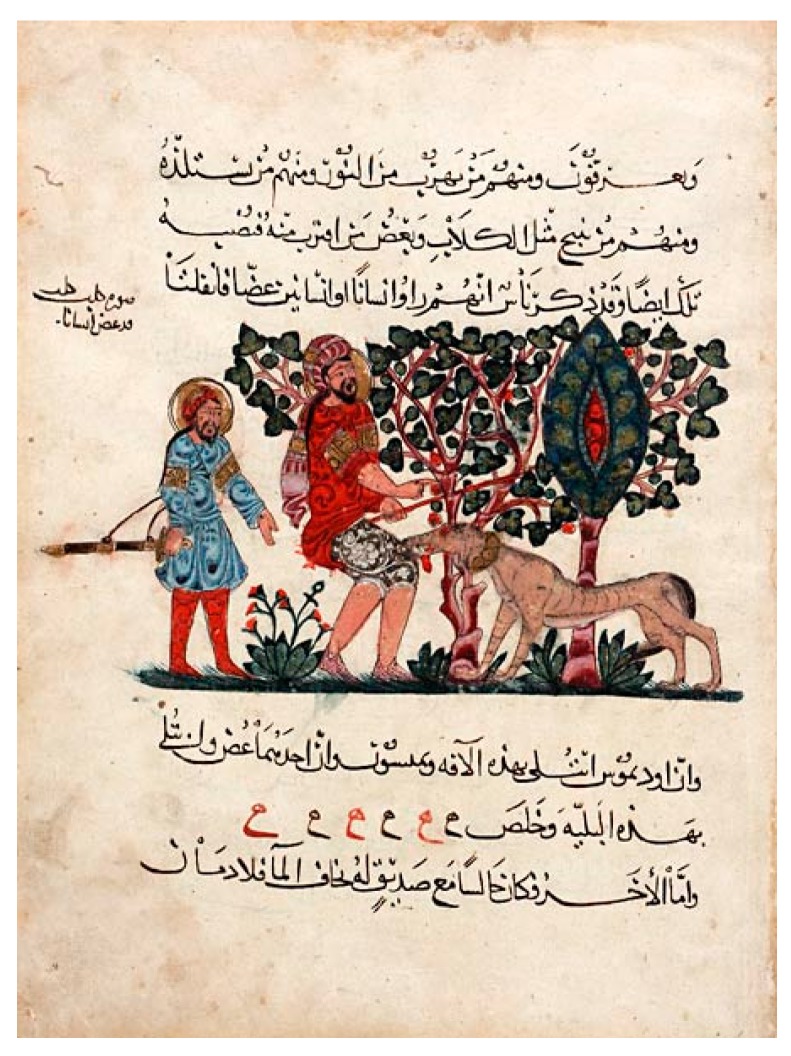
Outdoor scene with a mad dog biting a man. Folio from the ‘Kitab al-Hashaish’, an Arabic translation of the *Materia medica* by Dioscorides (ca. 40–90 C.E.) copied by Abdallah ibn al-Fadl, Baghdad, A.H.621/1224 A.D. Freer Gallery of Art and Arthur M. Sackler Gallery, Smithsonian Institution, Washington, D.C.: Purchase—Charles Lang Freer Endowment, F 1953.91.

The list continues with great mediaeval practitioners of medicine and botany, in Europe and elsewhere [19,28,31,33]. Despite religious antagonism against dogs, considered unclean, and recommendations for their containment [40] the mediaeval Middle East was rife with stray dogs [26,27,46]. Works by Mohammad-e Zakariā-ye Rāzi (Rhazes) [47,48], Ibn-Sīnā (Avicenna) [47,49], Moshe ben Maimon a.k.a. Mūsā ibn Maymūn (Maimonides) [27,29] and many others [32], all discuss or refer to dog-mediated rabies (Figure 5). Authors continued to accurately describe the disease in animals or humans, including the notion of paralytic rabies [40], the absence of hydrophobia in rabid dogs [40], or of a—in some cases considerable—delay [29] before symptoms onset of rabies in humans and its lamentable prognosis. No further remedy to clinically-declared rabies was identified.

At the end of the 13thC, Arnaldus de Villanova insisted on the importance of careful and thorough wound cleansing as prevention of rabies after a dog bite [50]. Bartholomew Glanville (mid-13thC) is said to have referred to a poison, “growing” and “multiplying” in bite wounds although this author found no primary source [40]. Prevention and treatment otherwise made no significant progress. Medical or surgical management delineated in Ancient Greece or Rome became increasingly tinted with religion. In Europe a miracle cure was deemed to be found at several specialized religious sites [51], such as the church of the village of Andage, renamed Saint-Hubert, where Louis I the Pious, one of Charlemagne’s sons and his successor, authorized the transfer of the eponymous saint’s thighbones in 826 CE. This abbey located near Liège, Belgium became a specialized center for rabies prevention. At the time, prevention before a bite took the form of applying a white-hot Key of Saint Hubert to dogs so they would not contract the disease [52,53]. An example of this amulet can be seen at http://www.webcitation.org/6os1x82Ty. Contrary to what was practiced in other reputed sites such as San Bellino [17], near present-day Rovigo in Italy, or in Saint-Tügen’s chapel in Primelin, France, this method must have been considered too cruel or too unreliable in humans bitten by suspected rabid animals. In humans, the preferred method of rabies prevention after a bite was based on incision of the forehead and implantation of threads from the Saint’s supposedly miraculous stole, accompanied by prayers and fasting [19,25,52,53,54]. In spite of Ambroise Paré—who after the siege of Turin in 1536 discontinued the practice of cauterization to heal wounds [55,56]—Dioscorides’ and Celsus’ cauterization approach remained widespread in the management of rabies risks well into the 19thC [31,57]. This may be because cauterization was performed to inactivate a “poison” and perhaps also because their work was never lost to practitioners in Europe in spite of the fall of the Roman Empire [58,59]. Patients, however, found little recourse should prevention fail: at Saint-Tügen chapel, patients with declared rabies were stifled between mattresses until the beginning of the 19thC.

## 4. After 1492: Emergence and Control

Rabies continued to concern populations and medical writers of the Renaissance. Julien Le Paulmier (1520–1588) wrote seven medical textbooks in all, one specifically on rabies [60,61].

The preventive practices at Saint-Hubert were condemned by the Sorbonne as superstitious in June 1671 [28] but remained in use in the Ardennes well into the 19thC [19,28,31,54]. The protective effect of thorough wound washing, and described anew in a publication dated 1796 cited by C. Ménécier, was by now well established among clinicians [62,63]. The converse was also true: the potentially deadly role of saliva was put to use by Polish-Lithuanian artillery general Kazimierz Siemienowicz (c. 1600–c. 1651), who in an early attempt at biological warfare, is said to have fired hollow shells containing saliva of rabid dogs in 1650 [64,65].

“Madstones”—bezoars or gallstones–thought to absorb or otherwise neutralize the agent of rabies were used extensively as amulets in mediaeval Europe and well into the 19thC by early European settlers in North America [31,66,67]. Dog-mediated rabies circulated in Europe, in Africa and in Asia [26,31,68]. Human deaths associated with bat bites were already identified in the mid-1500s in Latin America [69]. Although there were Nahuatl (Aztec) words for rabies and rabid dogs in what is now central Mexico, canine rabies was noticeably rare if not entirely absent from Central and South America [25,31,66,69]. Dog-mediated rabies, however, seems to have circulated more intensely and widely in both the Old and the New World after the landings of seafaring European conquerors and their dogs [19,25,68,70,71]. The 18^th^C was marked by intense rabies epizootics in the Americas and by the emergence of rabies on many islands of the Caribbean and the Indian Ocean [25,28,68,72]. Rabies became rampant among mongooses introduced in the Caribbean to eliminate rats pillaging sugar fields [73,74,75,76]. Colonial powers increasingly documented animal and human rabies cases in southern Africa in the 18th–19th Centuries [77].

Although circulation of rabies had reportedly increased, especially in Europe, great progress was being made in the prevention of dog bites in European cities [28,40]. Regulations for keeping dogs or for the containment of domestic dogs and elimination of stray dogs were passed in a city (Utrecht, Netherlands) in 1446 [78], in a Dutch province (Friesland) in 1714 [61], and in a country (Prussia) in 1787 [72,79,80]. A similar approach led to the successful elimination of dog-mediated rabies from Denmark, Norway and Sweden by 1826 [81]. Other long-known approaches including muzzling were implemented in other cities or territories [28,40,72]. In a 1793 communication, Samuel Bardsley proposed to quarantine local and imported dogs to “eradicate rabies from the British Isles” [40,82]. The decision to implement an international plan to control canine rabies was made at the 2nd International Veterinary Conference in Vienna on 21–27 August 1865. Cities and states legislated, integrating and applying early forms of what are now termed One Health principles [20,53].

The understanding of the physiopathology of rabies also evolved: in 1546, Girolamo Fracastoro hypothesized that rabies was transmitted by *semina* (“seeds”) present in the saliva [64,72,83,84]. Edward Topsell, translating Conrad Gessner’s work dated ca. 1555, mentions that rabies transmission is inconstant after the bite of a rabid dog [17]. Martin Lister added in 1698 that the risk of transmission varies according to the anatomical site of the bite [32], a notion comprehensively described by John Hunter in 1793 [85]. Joseph-Ignace Guillotin proposed in 1766 that biting dogs remain in 15-day observation to ascertain the risk of rabies transmission to a bite victim [40]. Van Swieten in 1775 declared saliva to be the source of rabies transmission and provides a clinical description in humans that remains relevant to date [86]. Hunter also spoke of many animals being, like humans, susceptible to rabies without being capable of transmitting the virus, and of that susceptibility being variable among species [85]. In 1776 and 1793, respectively, both Guillotin and Hunter proposed (dog bite) inoculation experiments to better understand the physiopathology of rabies, including in prisoners awaiting capital punishment [24,85,87]. Like Pasteur’s similar considerations plainly laid out in a letter to the Emperor of Brazil dated September 22, 1884, ten months before the post-exposure vaccination of Joseph Meister, these fortunately were never put into effect [87,88].

The understanding of post-bite rabies prevention in animals or in humans, however, still made no progress. Published on 17 June 1684, the first edition of *Medicina Curiosa*, the first English-language journal wholly dedicated to medicine, describes post-exposure prevention failure in a suspected human case of rabies acquired from a cat [89]. “Treatment” after a bite remained faith-based [90] or otherwise fanciful, based for example on applying hair of the biting dog (“hair of the dog”) to the wound [28,66] or omelets flavored with “dog-rose root” (*Rosa canina* or cynorrhodon, as already suggested by Pliny the Elder in the 1stC CE) [91,92,93,94,95]. The same was true outside Europe [96]. Suggested therapies—some even based on homeopathic approaches—were rightly criticized as ineffective [97]. The fact that rabies is not transmitted in all cases even after the bite of an evidently rabid dog or wolf contributed to the illusion that each of the many preventive “treatments” had been effective.

These are all too easily disparaged as ludicrous recommendations made by self-assured and pompous clinicians, steeped from old-wives’ remedies. They are, however, sure signs of desperate and all-out efforts by health providers of the time to save their patients from what to this day remains an intractable disease. Vigorous approaches continued to be used well into the mid-19thC: In 1830s London, children bitten by potentially rabid dogs still underwent surgery or cauterization of the wound [57] (still discussed by Babes in 1912 [72]). Patients with clinically declared rabies were plunged into cold water or hot oil as recommended by Celsus [31,86], or were later euthanized by being stifled between mattresses or made to bleed to death [17,90,98,99].

## 5. Pasteur and His Time

Around the turn of the 19thC, the scientific approach improved the understanding of the physiopathology and clinical epidemiology of rabies, which was remarkably summarized by Samuel Cooper in 1823 [100].

Much experimental work was done on the transmission of rabies [26,101,102,103]—and its prevention through the amputation (Helmann, cited in [72]) or immunization of animals [28,72,102,104]. In 1804 in Jena (in present-day Germany), Georg Zinke transmitted rabies experimentally (without a bite) by applying the saliva of rabid dogs to animals’ tissues [28,31,101,102,103,105]. The same was demonstrated in 1813 by Hugo Altgraf zu Salm-Reifferscheidt [106] and prior to 1814 by François Magendie and Gilbert Breschet, this time using saliva from a human rabies patient [107,108,109]. In 1805 in Turin, Francesco Rossi reported having experimentally transmitted rabies to dogs by inserting sciatic nerve segments of rabid cats into a fresh wound [110]. Clinicians progressively identified the seat of rabies infection in the midbrain [28,102] and nerve ending density was positively correlated with risks of infection and migration [72,111].

In the struggle pitting the microbial theory against spontaneous generation, subsequent experiments provided solid scientific evidence to support the long-suspected transmission of rabies by “filterable” infectious agents present in the saliva [101,102,104]: Magendie in 1842 suspected that the agent was not a poison but a “virus” capable of multiplying and developing in the host [112,113]. Magendie, then Casimir Davaine in 1872, experimented on virulence, increased by serial passage (but these were with septicemia and anthrax bacteria, not with viruses) [114,115,116]. In 1880, Edmond Nocard succeeded in separating saliva into two components, one non-infective and the other infective [117]. These agents were now considered to progressively ascend from the infected wound to the brain not through the blood but through the nerves—as initially hypothesized in 1879 but not established by Paul-Henri Duboué [118]—before diffusing centrifugally [31,72,102,112].

Resorting to nerve section as a means of prevention had been contemplated by George Hicks in 1807 [119]. Duboué—who communicated his findings to Louis Pasteur on 12 January 1881 [117]—also postulated that the rabies “virus” could be destroyed in situ or prevented from reaching the medulla oblongata [111]. This paved the way for the advent of post-exposure prophylaxis, based on the notion of taking advantage of the latency period and rapidly building the patient’s immunity through timely and adequate vaccination [102].

Variolation—the use of dried-out scabs containing attenuated smallpox virus to directly immunize against and prevent more severe smallpox–had been performed by intranasal insufflation in China since the 10thC, and inoculation was later extensively used in the Ottoman Empire [120,121]. This hazardous procedure was described by Emanuele Timoni in 1714 and subsequently experimented by Hans Sloane in English prisoners in 1722, after being championed by Mary Wortley Montagu [120,121,122,123,124,125,126]. Vaccination—the inoculation of virus causing much milder cowpox—to provide cross-immunization against smallpox had been pioneered by Benjamin Jesty in 1774, Peter Plett in 1790–1792 and Edward Jenner in 1796, perhaps based on John Fewster’s earlier work [121,127,128]. Putting John Hunter’s recommendations into practice, Eusebio Valli, an Italian physician, claimed to have carried out experimental infections and successfully immunized dogs by injecting the saliva of other dogs after submitting it to gastric juices of frogs in 1799. He claimed to have inoculated this mixture to at least two people in Pisa bitten by a suspected rabid dog and who did not contract rabies [24,129,130]. If confirmed, this would make Valli the initiator of the first attenuated vaccine and rabies vaccine, although the small numbers discussed and the absence of laboratory confirmation would not prove preventive effectiveness. Valli in 1816 made a fatal attempt at self-inoculation, not with rabies virus but with yellow fever, a few days after landing in Cuba to assist in an epidemic [26,131,132,133]. Although this author was unable to access original sources, Apollinaire Bouchardat, a pharmacist of the Veterinary Faculty in Lyons, is cited as having postulated in the 1850s that dogs could be immunized against rabies as a public health measure [134]. Available sources from 1882–1884 show Bouchardat discussing vaccination against infectious diseases, citing Pasteur’s work. In 1879, at the Veterinary school also in Lyons, rabies pioneer Pierre-Victor Galtier inoculated rabies to a rabbit through cutaneous injection, administered rabid dog saliva intravenously to a sheep which did not contract rabies but became immunized, theorized post-exposure prophylaxis and began experimenting on vaccination of dogs [102,135,136,137,138,139,140,141,142,143,144]. Henry Toussaint—another veterinarian—conducted research in Lyons on heat- and subsequently carbolic acid-attenuated anthrax vaccine in 1880 [144,145]. Paul Gibier from the Faculty of Medicine and the Muséum d’Histoire Naturelle de Paris, showed in 1883–1884 that the rabies virus lost virulence after dessication and that this approach could be used in humans [146,147].

It is in this already extremely rich and advanced research context that Louis Pasteur and his colleagues at the Ecole Normale Supérieure in Paris began to apply their systematic, rigorous and data-driven scientific methods to the study of rabies in December of 1880 [92,118,146]. Pasteur and his team had already developed an effective attenuated fowl cholera vaccine [148], were working on an attenuated anthrax vaccine and strove to apply their techniques to rabies—a much-feared and highly symbolic disease, albeit known to be controllable by veterinary measures alone [117,149]. An experimental model of rabies was developed by Paul Emilio (Emile) Roux in dogs inoculated after trepanation, and later in the noticeably more manageable rabbit [92,102]. A “fixed”, adapted, rabies virus strain of “exalted virulence” with shorter incubation times and unfailing transmission could then be selected through successive passage in the rabbit, thereby paving the way for an experimental and methodical approach. After discussing it in 1881 [150], Pasteur and his team endeavored in 1882 to develop a canine “vaccine” (thus named in honor of Jenner), using after 1884 the desiccation technique also developed by Emile Roux to attenuate this live, highly virulent virus [151,152,153,154]. Rabies virus attenuation was first validated by experiments which Pasteur and his team reported in 1884, documenting survival of dogs vaccinated by live, attenuated vaccine before viral challenge. The prototypal vaccine against rabies was first used as salvage therapy in humans presenting signs of declared clinical rabies, with rapid documented failure in at least one instance: that of the child Antoinette Poughon in late June 1885 [92,155]. The vaccine, however, was to meet resounding success in patients exposed to rabies virus but with yet no signs of declared infection.

**Figure 6:**
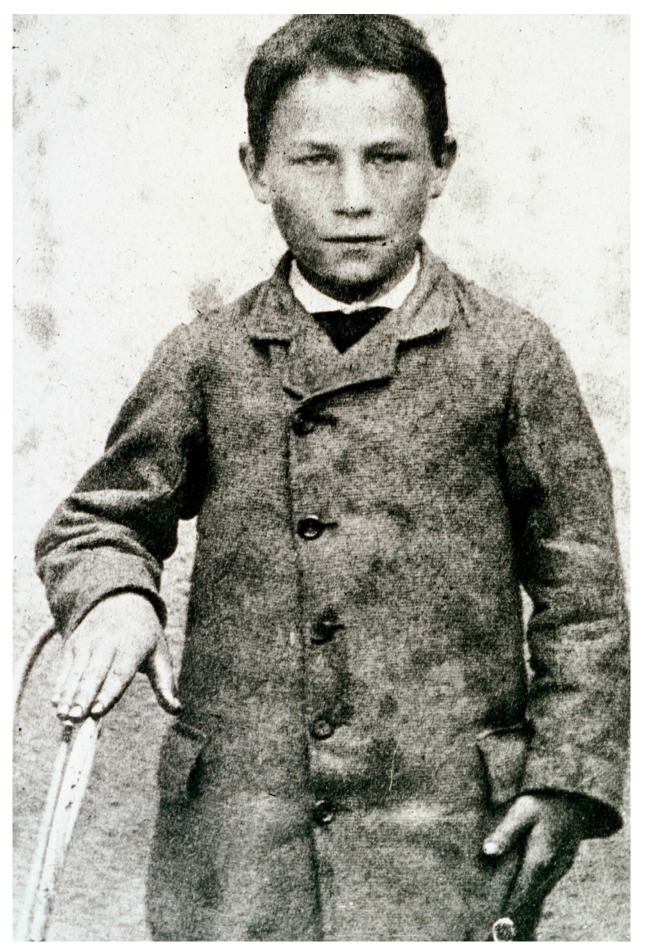
Joseph Meister in 1885, the first human to have received Pasteur’s live, attenuated rabies vaccine on July 6, 1885 (© Institut Pasteur-Musée Pasteur).

History remembers a 9-year-old schoolboy, Joseph Meister (Figure 6), attacked and bitten 14 times by Mr. Théodore Vonné’s dog while on an errand in Maisonsgoutte (Meissengott), in then German-occupied Alsace, on 4 July 1885 [152]. Joseph Meister suffered deep bites to the right hand and to the thighs and leg. The owner of the dog, Mr. Théodore Vonné (or Vone) also received one bite to the arm by the same dog before it was shot by the police; the bite being delivered through cloth (untorn) and leaving no wound, Mr. Vonné received no prophylaxis and survived [72,154,156,157]. Dr. Eugène Weber, a local medical doctor with a practice in nearby Villé, made a call to the Meister home that evening and cleansed the wound thoroughly with carbolic acid, 12 h after the attack [158]. As he waited in a café for the coach to return home, Vonné spoke of the event with clients and was told that Pasteur had developed prevention against rabies [158]. He went back to the Meister home to inform the family and Meister, accompanied by his mother and Vonné left the next morning by train and arrived in Paris on 6 July. Although a medical doctor, Emile Roux did not inject the vaccine into Meister. This may be because he was not regularly working in the laboratory at the time or had not practiced medicine for too long, but published sources agree it clearly was because Roux had unequivocally stated his concern that the rabies vaccination procedure developed in dogs was insufficiently tested and too risky to be used in humans [92,131,159,160]. Jacques-Joseph Grancher therefore administered subcutaneously the first doses of live attenuated rabies vaccine on 6 July 1885, at 8:00 PM in the presence of Louis Pasteur—who, as a chemist, was not authorized to perform injections—and Alfred Vulpian. The first injection was derived from the chord of an inoculated rabbit which died of rabies on 21 June (15 days earlier) [92]. Over the 10 following days, Joseph Meister received 12 additional doses of attenuated and progressively more virulent virus to quickly generate an immune response, in an attempt to beat the virus in a deadly race against time [19,33,72]. Meister survived.

This successful attempt was repeated in late October 1885 in a second case, that of a 15-year-old shepherd, Jean-Baptiste Jupille from Villers-Farlay, Jura, who sustained on October 14 a deep bite to the left and right hands after an attack by a furious dog [92,152]. Jupille was referred to Pasteur by the town mayor and received rabies post-exposure prophylaxis (PEP) in Paris from 20 to 30 October, 1885. Following Grancher’s accidental exposure to the attenuated vaccine during Jupille’s PEP, Adrien Loir and Eugène Viala became the two first humans to receive *pre*-exposure rabies vaccination [134]. Having become a laboratory assistant in the Pasteur team, Meister was also the first to receive rabies vaccine boosters when he underwent a—reportedly less well-tolerated—second PEP in 1890 after being bitten by a guinea pig with experimental rabies (M-T. Meister, personal communication, 16 May 2016).

For the very first time since its first recorded description 3800 years earlier, and despite some failures due mostly to delayed referral [92,154], clinicians now had a proven and effective means of rabies prevention in humans. This led to Louis Pasteur’s laboratory at École Normale Supérieure, Paris routinely offering PEP services. Around one year after the first PEP, L. Pasteur in August 1886 reported 3 (0.2%) deaths (whether the case of Louise Pelletier is included among these deaths is unclear) among 1235 PEP recipients [92], while another source speaks of 21 (1.0%) deaths among 1986 recipients (including one from Bombay, India) by 22 August of that same year [161]. In 1887, Vulpian documented 12 (0.7%) deaths among 1726 PEP recipients, for an expected number of approximately 264 (15.3%) rabies deaths if PEP had not been administered [162].

The rabbit cord used in the Pasteur vaccination protocol was known to preserve its virulence despite preservation in carbolic acid [152]. It was, however, not stabilized and therefore not usable outside Paris unless “transported” by/in inoculated rabbits. Patients therefore had to travel to access PEP, in some cases across continents or oceans [163,164]. After PEP spared the lives of 16 of 19 Russian patients who came to Paris from Smolensk after being attacked by a rabid wolf [165], Elie Metchnikoff was named director of the first center established specifically to produce rabies vaccine (which benefited from Louis Pasteur’s support) and implemented the “Pasteur treatment” in Odessa in June 1886, [166,167,168]. The not-for-profit, non-governmental Institut Pasteur Foundation was incorporated in France by a decree on 4 June 1887. The Institut Pasteur itself was built and inaugurated on 14 November 1888, after an unprecedented national and international movement and fundraising campaign to further disseminate the technique and to pursue research [19,118,163].

## 6. Modern Developments

**Figure 7:**
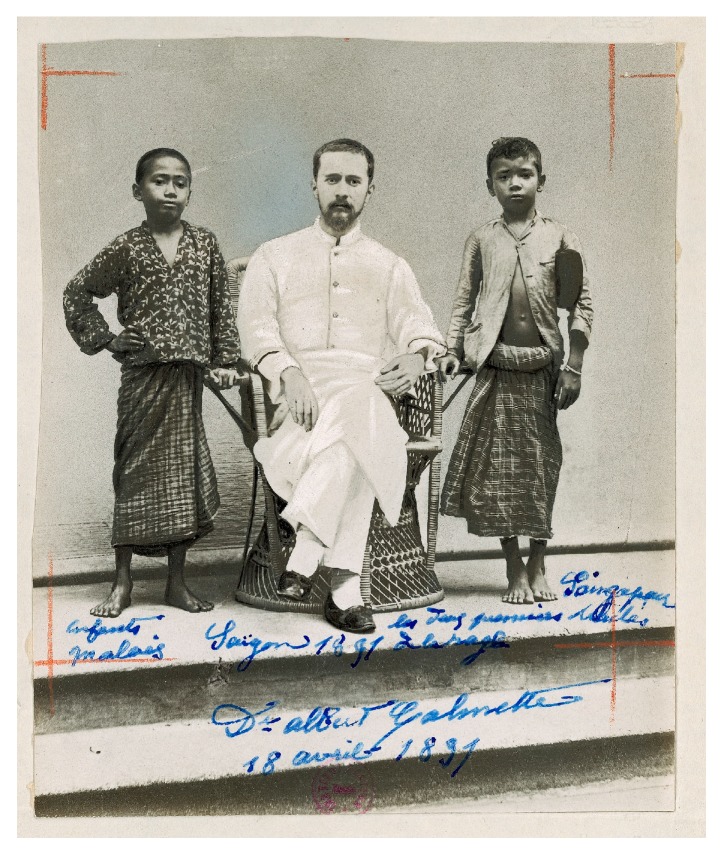
Albert Calmette and the first two patients to receive rabies PEP in Asia (excluding the Russian Empire), Africa or Latin America, 18 April 1891. The handwritten legend indicates that these were Malay children referred from Singapore (© Institut Pasteur-Musée Pasteur).

Over the decades that followed the development of PEP by Pasteur and his team, many rabies prevention centers or “Pasteur institutes”—some affiliated with the Institut Pasteur in Paris, most not [167]—appeared across the Old and the New World. In 1909 there were 75 such centers worldwide, including in then Indochina [72,169]. These centers cultured in vivo then attenuated highly virulent rabies virus (RABV) locally. In Saigon, animal bite victims received PEP as early as 1891, becoming the first to receive rabies PEP in Asia, Africa or Latin America (Figure 7) [170,171,172,173,174]. This was facilitated by RABV preservation techniques in glycerin, also developed by Emile Roux and Albert Calmette [30,118], which no longer made uninterrupted sequences of RABV inoculation to successive unfortunate rabbits every ten days a requirement to preserve live virus.

Post-exposure prophylaxis biologicals and procedures were improved in the ensuing decades. The rabies vaccine was further refined by Emile Roux [170,175], Victor Babes [72], Follen Cabot [176], Claudio Fermi, Endre Högyes [177] and especially David Semple [177,178,179]. Babes and M. Lepp in 1889 first described immunity as a correlate of vaccine response and protection, discussed inactivated rabies vaccines and experimentally demonstrated protection of animals by antiserum in 1891 [72,177,180]. Solutions of attenuated virus mixed with serum immunoglobulin were experimented at the Pasteur Institute as early as 1902 [169]. Rabies antiserum was administered in humans to interrupt replication of the virus in bite wounds by Fermi in 1911 and the use of rabies-specific immunoglobulin was generalized in the 1950s [31,181,182,183,184,185,186]. Monoclonal antibodies (produced either in animals or by yeasts or plants) are now being developed to replace unaffordable equine—let alone human—rabies immunoglobulin (RIG), so far with mixed but promising results [187,188,189,190,191,192,193,194].

Semple’s killed-virus vaccine, developed in 1911 at the Pasteur Institute in Kasauli, India, using sheep brain tissue, remained the most used worldwide into the 2000s. Although the vaccine had limited immunogenicity and required a tedious protocol (and was painful, as experienced first-hand by the author in West Africa as a child in the 1970s) it was affordable and for decades saved countless human lives, especially in the developing world. The League of Nations’ health organization’s bulletin reported 115,859 PEP worldwide recipients in 1932-May 1934 among whom 439 (0.4%) were considered to have died of rabies [195]. After initial attempts at the Institut Pasteur in 1913 [196], the rabies virus was successfully cultured in vitro through several passages in 1936 [197]. In the 1960s, harvests of RABV grown in tissue cultures became increasingly pure [198] and normative methods were developed to standardize the potency of the various vaccines [31,199,200]. Vaccines were developed on suckling mouse brains [201] or on duck or chicken embryos [202], until the advent of new, highly antigenic, better-tolerated cell-culture vaccines [33,203,204,205,206]. This allowed for the tedious Pasteur then Semple protocols to be progressively replaced by the shorter Essen and Zagreb protocols [207]. An oral vaccine was developed for wildlife in 1971 [208]. Through canine population regulations and control, rabies was eliminated from cities in the industrialized world and elsewhere, including Shanghai in 1949 and Malaya in the early 1950s [25,209].

Research on the rabies virus itself made rapid advances. In 1903, Adelchi Negri described the first RABV-neuron interaction and Lina Luzzani-Negri described its diagnostic value in infection with “street” rabies virus [210,211,212]. The rabies virus itself was first observed by electron microscope in the early 1960s [213,214,215]. The molecules produced by RABV (transcriptional mapping) were described in 1978 [216] and the viral genes which code for them were sequenced in their entirety in 1988 [217]. Direct and indirect diagnostic methods were developed to reliably confirm infection and antibody protection [200,218,219].

These advances led to the validation of rabies vaccine effectiveness, of shorter and dose-sparing regimens and of the equivalence of the intradermal vaccination route [220,221,222]. It also enabled the identification of nonfatal cases of RABV infection in animals [223,224,225,226] and in humans [227,228,229]. Human survivors of clinical rabies were first documented, mostly in the New World following bat exposure [230,231,232,233,234,235,236].

Whether or not these survived thanks to attempted treatment remains hotly debated [33,237,238,239]. Despite our dramatic advancements in the knowledge and prevention of rabies, and with a handful of exceptions to date [229], all documented patients with clinically-declared rabies have died within a few hours or days. Efforts to test some traditional medicines, in Ethiopia for example, have failed [240]. Antivirals are currently being explored as a therapeutic resource, so far with little success [241].

## 7. Conclusions

Our understanding of the mechanisms and primary and secondary prevention of rabies in animals and in humans has profoundly changed since the Laws of Eshnunna were introduced by one of the earliest known civilizations. Yet despite this, and great progress in symptomatic management of encephalitic patients, clinicians caring for animals or patients with symptomatic rabies remain as powerless today as they were 4000 years ago. Rabies remains today the most lethal disease known to man and this author is not aware of any other disease for which—once the disease is declared—modern medicine has offered no tangible improvement. We wait in hope for researchers to identify antiviral agents capable of controlling progression of clinically-declared rabies.

Rabies became a neglected disease when it was eliminated from Europe and North America. It is emerging in some island territories and remains uncontrolled in most of the developing world, where surveillance of dog bites, rabies exposures (syndromic or laboratory-confirmed) or rabies deaths, is poor [242,243]. The prevention of human rabies deaths in the 21stC still rests on tools and strategies developed in the 19thC: Effective primary prevention of animal bites and responsible dog ownership as delineated by Fleming (in 1872) [28]; canine vaccination as proposed by H. Bouley (in 1884) [72] and timely and effective rabies post-exposure prophylaxis (developed by Pasteur and his team and first administered in 1885).

An estimated total of 59,000 humans die of rabies each year, more than twice the estimated 28,600 deaths caused by the tragic 2014–2016 Ebola outbreak in West Africa [244,245]. The World Health Organization, the World Organization for Animal Health and the Food and Agriculture Organization of the United Nations are currently spearheading an effort to eliminate dog-transmitted rabies worldwide by 2030 [246]. While we strive for all dogs to be vaccinated, a major effort is urgently needed to make the time-proven and well-tolerated vaccine (and immunoglobulin) geographically and financially accessible in a timely way to those people who remain the most vulnerable to rabies: the rural populations of developing countries [247,248].

## References

[1] Dayan T. (1994). Early domesticated dogs of the Near East. J. Archaeol. Sci..

[2] Thalmann O., Shapiro B., Cui P., Schuenemann V.J., Sawyer S.K., Greenfield D.L., Germonpré M.B., Sablin M.V., López-Giráldez F., Domingo-Roura X. (2013). Complete mitochondrial genomes of ancient canids suggest a European origin of domestic dogs. Science.

[3] Armelagos G. (1997). Disease, Darwin, and medicine in the third epidemiological transition. Evol. Anthropol. Issues News Rev..

[4] Zuckerman M.K., Harper K.N., Barrett R., Armelagos G.J. (2014). The evolution of disease: Anthropological perspectives on epidemiologic transitions. Glob. Health Action.

[5] Greger M. (2007). The human/animal interface: Emergence and resurgence of zoonotic infectious diseases. Crit. Rev. Microbiol..

[6] Rashidi J.S. (2011). Paleoepidemiology of Mesopotamia and the ancient Near East: The impact of zoonotic diseases and population demographics on infectious disease patterns. Ph.D. Thesis.

[7] (2014). Eshnunna. Wikipedia Free Encycl..

[8] Roth M.T., Hoffner H.A., Michalowski P. (1997). Law collections from Mesopotamia and Asia Minor: Writings from the Ancient World.

[9] Yuhong W. (2001). Rabies and rabid dogs in Sumerian and Akkadian literature. J. Am. Orient. Soc..

[10] Goetze A. (1965). The laws of Eshnunna. Annu. Am. Sch. Orient. Res..

[11] Abusch I.T., Toorn K.V.D. (1999). Mesopotamian Magic: Textual, Historical, and Interpretative Perspectives.

[12] Sigrist M., Marks J.H., Good R.M. (1987). On the bite of a dog. Love and Death in the Ancient near East: Essays in Honor of Marvin H. Pope.

[13] Veldhuis N. (1993). An Ur III Incantation against the bite of a snake, a scorpion, or a dog. Zeitschrift für Assyriologie und Vorderasiatische Archäologie.

[14] Geller M.J. (2003). Ur III Incantations from the Frau Professor Hilprecht-Collection, Jena.

[15] Black J., Green A. (2004). Gods, Demons and Symbols of Ancient Mesopotamia: An Illustrated Dictionary: Jeremy Black, Anthony Green: 9780292707948: Amazon.com: Books.

[16] Ornan T. (2004). The Goddess Gula and her dog. IMSA.

[17] Swabe J., King A.A., Fooks A.R., Aubert M., Wandeler A.I. (2004). Chapter 22: Folklore, perceptions, science and rabies prevention and control. Historical Perspective of Rabies in Europe and the Mediterranean Basin.

[18] Fustel de Coulanges (2009). La cité antique.

[19] Wasik B., Murphy M. (2013). In the beginning. Rabid: A Cultural History of the World’s Most Diabolical Virus.

[20] Menezes R. (2008). Rabies in India. CMAJ Can. Med. Assoc. J..

[21] Bhishagratna K.K.L. (1907). Chapter VI. An English Translation of the Sushruta Samhita, Based on Original Sanskrit Text by Susruta.

[22] Zhang Y.-Z., Xiong C.-L., Xiao D.-L., Jiang R.-J., Wang Z.-X., Zhang L.-Z., Fu Z.F. (2005). Human rabies in China. Emerg. Infect. Dis..

[23] Adedeji A.O., Okonko I.O., Eyarefe O.D., Adedeji O.B., Babalola E.T., Ojezele M.O., Nwanze J.C., Amusan T.A. (2010). An overview of rabies—History, epidemiology, control and possible elimination. Afr. J. Microbiol. Res..

[24] Bellini F., Fossati P., Liverini A. (2009). L’evoluzione della rabbia attraverso i secoli. Rassegna Dirit. Legis. E Med. Leg. Vet..

[25] Baer G.M., Wunner W.H., Jackson A.C. (2010). The history of rabies. Rabies: Scientific Basis of the Disease and Its Management.

[26] Blancou J., King A.A., Fooks A.R., Aubert M., Wandeler A.I. (2004). Chapter 2: Rabies in Europe and the Mediterranean Basin: From Antiquity to the 19th Century. Historical Perspective of Rabies in Europe and the Mediterranean Basin.

[27] Yakobson B.A., David D., Aldomy F., King A.A., Fooks A.R., Aubert M., Wandeler A.I. (2004). Chapter 13: Rabies in Israel and Jordan. Historical Perspective of Rabies in Europe and the Mediterranean Basin.

[28] Fleming G. (1872). Rabies and Hydrophobia: Their History, Nature, Causes, Symptoms, and Prevention.

[29] Rosner F. (1974). Rabies in the Talmud. Med. Hist..

[30] Schneider M.C., Santos-Burgoa C. (1994). Treatment of human rabies: A summary of its history. Rev. Saúde Pública.

[31] Steele J.H., Fernandez P.J., Baer G.M. (1991). The Natural History of Rabies, 2nd Edition. The Natural History of Rabies.

[32] Moreau R., Rosset R. (1985). La rage de l’Antiquité au Siècle des Lumières. Pasteur et la Rage.

[33] Jackson A.C., Jackson A.C. (2013). Chapter 1—History of rabies research. Rabies.

[34] Neville J., King A.A., Fooks A.R., Aubert M., Wandeler A.I. (2004). Chapter 1: Rabies in the Ancient World. Historical Perspective of Rabies in Europe and the Mediterranean Basin.

[35] Wang Z.G., Chen P., Xie P. (1999). History and Development in Traditional Chinese Medicine.

[36] On the mad dog and the dog diseased; how they are to be kept, and cured. http://www.avesta.org/vendidad/vd13sbe.htm.

[37] Cardiff R.D., Ward J.M., Barthold S.W. (2008). “One medicine—one pathology”: Are veterinary and human pathology prepared?. Lab. Investig. J. Tech. Methods Pathol..

[38] Schwabe C.W. (1991). History of the scientific relationships of veterinary public health. Rev. Sci. Tech. Int. Off. Epizoot..

[39] (1941). Lucius Junius Moderatus Columella Book. On Agriculture, with a Recension of the Text and an English Translation.

[40] Blancou J. (1994). Early methods for the surveillance and control of rabies in animals. Rev. Sci. Tech. Int. Off. Epizoot..

[41] Griffith F.L., Griffith F.L., Thompson H. (1904). The Demotic Magical Papyrus of London and Leiden.

[42] (1935). Aulus Cornelius Celsius Book V—Chapter 27. De Medicina.

[43] Köckerling F., Köckerling D., Lomas C. (2013). Cornelius Celsus—Ancient encyclopedist, surgeon–scientist, or master of surgery?. Langenbecks Arch. Surg..

[44] (1959). Claudius Aelianus (Aelian) XIV. On the Characteristics of Animals.

[45] Allbutt T.C. (1921). Greek Medicine in Rome: The Fitzpatrick Lectures on the History of Medicine Delivered at the Royal College of Physicians of London in 1909–1910, with Other Essays.

[46] Menache S. (1997). Dogs: God’s worst enemies?. Soc. Anim..

[47] Hatami H. (2012). History of rabies in traditional medicine’s resources and Iranian research studies: On the occasion of the World Rabies Day (28 September 2012). Int. J. Prev. Med..

[48] Tadjbakhsh H. (1994). Traditional methods used for controlling animal diseases in Iran. Rev. Sci. Tech. Int. Off. Epizoot..

[49] Dalfardi B., Esnaashary M.H., Yarmohammadi H. (2014). Rabies in medieval Persian literature—The Canon of Avicenna (980–1037 AD). Infect. Dis. Poverty.

[50] Arnaldi de Villanova Capitulum. XIII (1485). De morsu canis rabidi. Breviariuz pratice.

[51] Théodoridès J., Lépine P. (1986). Histoire de la Rage: Cave Canem.

[52] Guitard E.-H., Amblard É. (1960). L’histoire merveilleuse de la clef de saint Hubert. Rev. Hist. Pharm..

[53] Bazin H. (2007). Saint Hubert, guérisseur de la rage de l’homme et des animaux. Bull. Soc. Hist. Méd. Sci. Vét..

[54] Schayes A.-G.-B. (1834). Essai Historique sur les Usages, les Croyances, les Traditions, les Cérémonies et Pratiques Religieuses et Civiles des Belges Anciens et Modernes.

[55] Donaldson I. Ambroise Paré’s accounts of new methods for treating gunshot wounds and burns. http://www.jameslindlibrary.org/articles/ambroise-pares-accounts-of-new-methods-for-treating-gunshot-wounds-and-burns/.

[56] Paré A. (1652). Les oeuvres d’Ambroise Paré.

[57] Pemberton N., Worboys M. (2007). Mad Dogs and Englishmen: Rabies in Britain 1830–2000: Rabies in Britain, 1830–2000.

[58] (2015). Pedanius Dioscorides. Wikipedia Free Encycl..

[59] De Vos P. (2010). European Materia Medica in historical texts: Longevity of a tradition and implications for future use. J. Ethnopharmacol..

[60] Le Paulmier de Grantmesnil J.I. (1578). Palmarii Constantini de morsu caninis rabidi et hydrophobia, liber. Iulii Palmarii, Constantini, medici Parisiensis, De Morbis Contagiosis Libri Septem: Ad Amplissimum Senatum Parisiensem; Cum Indice Gemino.

[61] Nutton V. (2011). Understanding contagious diseases: Baillou’s notes on Julien Le Paulmier’s De morbis contagiosis. Med. Storia.

[62] (1796). Allan Observations sur la rage. Recl. Périodique Société Santé Paris Vendémiaire Brum. V.

[63] Ménécier C. (1864). Notice sur la rage: avec un projet nouveau de police sanitaire sur la race canine: présenté à Son Excellence M. le ministre de l’agriculture et du commerce / par le docteur Charles Ménécier,....

[64] DiMarco V. (2014). The Bearer of Crazed and Venomous Fangs.

[65] Ramasamy S., Liu C., Tran H., Gubala A., Gauci P., McAllister J., Vo T. (2010). Principles of antidote pharmacology: an update on prophylaxis, post-exposure treatment recommendations and research initiatives for biological agents. Br. J. Pharmacol..

[66] Kumar P.D. (2009). Rabies.

[67] Mike Nichols Nineteenth-Century Medicine: “Sir, the Madstone Will See You Now”. http://www.webcitation.org/6os1vFBQS.

[68] Nadin-Davis S.A., Bingham J., King A.A., Fooks A.R., Aubert M., Wandeler A.I. (2004). Chapter 19: Europe as a source of rabies for the rest of the world. Historical Perspective of Rabies in Europe and the Mediterranean Basin.

[69] Vos A., Nunan C., Bolles D., Müller T., Fooks A.R., Tordo N., Baer G.M. (2011). The occurrence of rabies in pre-Columbian Central America: an historical search. Epidemiol. Infect..

[70] Troupin C., Dacheux L., Tanguy M., Sabeta C., Blanc H., Bouchier C., Vignuzzi M., Duchene S., Holmes E.C., Bourhy H. (2016). Large-scale phylogenomic analysis reveals the complex evolutionary history of rabies virus in multiple carnivore hosts. PLOS Pathog..

[71] Schwartz G.R. (2008). The History and Development of Caravels. Master’s Thesis.

[72] Babes V. (1912). Traité de la Rage.

[73] Barun A., Hanson C.C., Campell K.J., Simberloff D. (2011). A review of small Indian mongoose management and eradications on islands. IUCN.

[74] World Health Organization (2004). WHO Expert Consultation on Rabies (First Report).

[75] Tierkel E.S., Arbona G., Rivera A., de Juan A. (1952). Mongoose rabies in Puerto Rico. Public Health Rep..

[76] Styczynski A. (2017). Human Rabies—Puerto Rico, 2015. MMWR Morb. Mortal. Wkly. Rep..

[77] Brown K. (2011). Travelers and Doctors. The mystery of rabies in colonial South Africa. Mad Dogs and Meerkats: A History of Resurgent Rabies in Southern Africa.

[78] Van der Monde N. (1839). Bepaling op Het Houden van Honden, Binnen Utrecht, in Het Jaar 1446, Des Woensdags na St. Michielsavont. Tijdschrift voor Geschiedenis, Oudheden en Statistiek van Utrecht: Met Naamlijst der Geborenen, Ondertrouwden en Overledenen Binnen Utrecht en Voorsteden.

[79] Germany P. (1787). Extrakt aus dem Edict wegen des Tollwerdens der Hunde vom 20 Februar 1787.

[80] Müller W., Cox J., Müller T. (2004). Rabies in Germany, Denmark and Austria. Historical Perspective of Rabies in Europe and the Mediterranean Basin—A Testament to Rabies by Dr Arthur A. King.

[81] Evans A.S., Kaslow R.A. (1997). Viral Infections of Humans: Epidemiology and Control.

[82] Brockbank E.M., Edward M. (1904). Sketches of the Lives and Work of the Honorary Medical Staff of the Manchester Infirmary, from Its Foundation in 1752 to 1830 When It Became the Royal Infirmary.

[83] Riddell W.R. (1923). Hydrophobia: Four Centuries Ago. Public Health J..

[84] Meunier L. (1893). Chapitre X—De la Rage (Caput X—De Rabie). Les Trois Livres de Jérôme Fracastor sur la Contagion, les Maladies Contagieuses et Leur Traitement.

[85] Hunter J. (1793). Observations, and Heads of Inquiry, on Canine Madness, drawn from the Cases and Materials collected by the Society, reflecting that Disease. Transactions of a Society for the Improvement of Medical and Chirurgical Knowledge.

[86] Swieten G.V. (1774). An Abridgement of Baron Van Swieten’s Commentaries upon the Aphorisms of ... Herman Boerhaave Concerning the Knowledge and Cure of Diseases.

[87] Soubiran A., Théodoridès J. (1982). Guillotin et la rage: Un Mémoire inédit. Hist. Sci. Medicales.

[88] Arboleda-Flórez J. (2005). The ethics of biomedical research on prisoners. Curr. Opin. Psychiatry.

[89] Colman E. (1999). The first English medical journal: Medicina Curiosa. Lancet.

[90] (1895). Les protecteurs contre la rage. Oeuvres Complètes de Mgr. X. Barbier de Montault, Prélat de la Maison de Sa Sainteté.

[91] Andry C.L.F. (1780). Recherches sur la Rage.

[92] Debré P. (1997). Louis Pasteur.

[93] Withey D.A. Mad dog (bites) and Englishmen: early-modern remedies for hydrophobia. Dr Alun Withey.

[94] Diderot D., le Rond d’Alembert J.R. (1765). Encycl. Ou Dict. Raison. Sci. Arts Métiers Etc.

[95] Berkenhout J. (1783). An Essay on the Bite a Mad Dog: In Which the Claim to Infallibility of the Principal Preservative Remedies against the Hydrophobia Is Examined.

[96] Pankhurst R. (1970). The history and traditional treatment of rabies in Ethiopia. Med. Hist..

[97] Bouchardat A. (1852). Rapport général (...) sur divers remèdes proposés pour prévenir ou pour combattre la rage. Bull. Acad. Natl. Méd..

[98] Magendie F. (1838). Course of the lectures on the blood and on the changes which it undergoes during disease, delivered at the College de France in 1837-8—Lecture IX. Lancet.

[99] Buisson F. (1836). Traité sur L’hydrophobie ou Rage. Moyen de Prévenir et de Guérir Cette Maladie par BUISSON.

[100] Cooper S. (1823). A Dictionary of Practical Surgery: Comprehending All the Most Interesting Improvements, from the Earliest Times down to the Present Period; an Account of the Instruments, Remedies and Applications Employed in Surgery; the Etymology and Signification of the Principal Terms; ... Forming Together a “catalogue Raisonné” of Surgical Literature ....

[101] Hertwig K.H. (1829). Contribution towards a better knowledge of rabies canina [Beiträge zur nähern Kentniss der Wuthkrankheit, etc.]. The Edinburgh Medical and Surgical Journal ....

[102] Roux E.P.P. (1883). Des Nouvelles Acquisitions sur la Rage. Thèse de Médecine.

[103] Pearce J.M.S. (2002). Louis Pasteur and rabies: A brief note. J. Neurol. Neurosurg. Psychiatry.

[104] Rosset R. (2003). Pasteur et les vétérinaires. Bull. Soc. Fr. Hist. Méd. Sci. Vét.

[105] Zinke G.G. (1804). Neue Ansichten der Hundswuth; ihrer Ursachen und Folgen, nebst einer sichern Behandlungsart der von tollen Thieren gebissenen Mensch ....

[106] Baer G.M. (1975). The natural history of rabies.

[107] Théodoridès J. (1978). Dupuytren et la rage. Hist. Sci. Médicales.

[108] Breschet G. (1840). Recherches expérimentales relatives au mode de transmission de la rage. Archives générales de médecine.

[109] Wright S. (1842). The physiology and pathology of the saliva. The Lancet.

[110] Vassali-Eandi A.M. (1809). Notice des ravaux de la classe des sciences physiques et mathématiques. Mémoires de l’Académie Impériale des Sciences, Littérature et Beaux-Arts de Turin pour les Années 1805–1808. Sciences Physiques et Mathématiques.

[111] Duboué P.H., Royal College of Physicians of Edinburgh (1879). De la Physiologie Pathologique et du Traitement Rationnel de la Rage: Suite D’études de Pathogénie.

[112] Théodoridès J. (1983). Magendie et la pathologie infectieuse. Hist. Sci. Medicales.

[113] Magendie F. (1862). Leçons faites au Collège de France pendant le semestre d’hiver (1851–1852) par M. Magendie, Recueillies et Analysées par le Dr V.A. Fauconneau Dufresne.

[114] Davaine C. (1872). Recherches sur quelques questions relatives à la septicémie. Bull. Acad. Natl. Méd..

[115] Théodoridès J. (1966). Casimir Davaine (1812–1882): A precursor of Pasteur. Med. Hist..

[116] Wrotnowska D. (1976). Pasteur et Davaine d’après des documents inédits. Hist Sci. Med..

[117] Moreau R., Rosset R. (1985). Notes sur Pasteur et la rage: A propos de quelques lettres ou documents inédits. Pasteur et la Rage.

[118] Perrot A., Schwartz M. (2013). Pasteur et ses Lieutenants: Roux, Yersin et les autres.

[119] Hicks G. (1807). Mr. Hicks, on hydrophobia. Lond. Med. Phys. J..

[120] Weiss R.A., Esparza J. (2015). The prevention and eradication of smallpox: A commentary on Sloane (1755) “An account of inoculation”. Philos. Trans. R. Soc. B Biol. Sci..

[121] Fenner F., Henderson D.A., Arita I., Jezek Z., Ladnyi I.D. (1988). Early Efforts at Control: Variolation, Vaccination, and Isolation and Quarantine. Smallpox and Its Eradication.

[122] Gross C.P., Sepkowitz K.A. (1998). The myth of the medical breakthrough: smallpox, vaccination, and Jenner reconsidered. Int. J. Infect. Dis. IJID Off. Publ. Int. Soc. Infect. Dis..

[123] Pead P.J. (2003). Benjamin Jesty: New light in the dawn of vaccination. Lancet.

[124] Pead P.J. (2006). Benjamin Jesty: The first vaccinator revealed. Lancet.

[125] Boylston A. (2012). The origins of inoculation. J.R. Soc. Med..

[126] Hawkins S.A. (2010). Sir Hans Sloane (1660–1735): His life and legacy. Ulster Med. J..

[127] Plett P.C. (2006). Peter Plett and other discoverers of cowpox vaccination before Edward Jenner. Sudhoffs Arch..

[128] Thurston L., Williams G. (2015). An examination of John Fewster’s role in the discovery of smallpox vaccination. J.R. Coll. Physicians Edinb..

[129] Valli E. (1805). Sulla peste di Costantinopoli del 1803 giornale del dottore Eusebio Valli cittadino fiorentino....

[130] Castiglioni A. (1937). VALLI, Eusebio. Encicl. Ital. Sci. Lett. Ed Arti.

[131] Bazin H. (2008). L’Histoire des Vaccinations.

[132] Denis A.L. (2007). Disease and Society in Colonial Cuba, 1790–1840. P.h.D. Thesis.

[133] Frank J. (1835). Encyclopédie des Sciences Médicales: Ou Traité Général, Méthodique et Complet des Diverses Branches de L’art de Guérir. Médecine. Pathologie Médicale/Par Joseph Frank. Deuxième Division.

[134] Rotivel Y., Goudal M., Perrin P., Tordo N. (2002). Une histoire de la vaccination contre la rage. Virologie.

[135] Galtier P. (1879). Etudes sur la rage. Note de M. Galtier, présentée par M. Bouley. C. R. L’Académie Sci..

[136] Galtier P. (1881). Les injections de virus rabique dans le torrent circulatoire ne provoquent pas l’éclosion de la rage et semblent conférer l’immunité. La rage peut être transmise par l’ingestion de la matière rabique (Note présentée par M. Bouley). Rev. Anal. Sociétés Savantes Fr. Létranger Académie Sci..

[137] Smith K.A. (2012). Louis Pasteur, the father of immunology?. Immunol. Mem..

[138] Théodoridès J. (1973). Quelques grands précurseurs de Pasteur. Hist Sci Med.

[139] Rosset R., Rosset R. (1985). Pierre Victor Galtier: Professeur à l’Ecole Vétérinaire de Lyon, précurseur de la vaccination antirabique. Pasteur et la Rage.

[140] Galtier P. (1881). Transmission du virus rabique (Observations à l’occasion du procès-verbal). Bull Acad Méd.

[141] Lombard M., Pastoret P.P., Moulin A.M. (2007). A brief history of vaccines and vaccination. Rev. Sci. Tech. Int. Off. Epizoot..

[142] Lepine P. (1969). Galtier and research on rabies. Bull. Acad. Natl. Med..

[143] Mérieux C. (1979). [1879–1979. It is now one hundred years since Victor Galtier, a professor of Veterinary School in Lyon, presented a paper on the prophylaxis of rabies to the Academy of Sciences]. Bull. Acad. Natl. Med..

[144] Williams E. (2010). The forgotten giants behind Louis Pasteur: Contributions by the veterinarians Toussaint and Galtier. Vet. Herit. Bull. Am. Vet. Hist. Soc..

[145] Toussaint H. (1880). Note contenue dans un pli cacheté et relative à un procédé pour la vaccination du mouton et du jeune chien (Note de Toussaint présentée par M. Bouley). C. R. Acad. Sci..

[146] Vignal M.W. (1886). Report on M, Pasteur’s Researches on Rabies and the Treatment of Hydrophobia by Preventive Inoculation. Br. Med. J..

[147] Gibier P. (1884). Recherches Expérimentales sur la Rage et sur son Traitement/Par Paul Gibier,...; avec Une Préface de M.H. Bouley, ....

[148] Pasteur L. (1880). De l’atténuation du virus du choléra des poules. C. R. Acad. Sci..

[149] Pasteur L., Chamberland C.E., Roux E.P.P. (1881). Sur la vaccination charbonneuse. C. R. Acad. Sci..

[150] Pasteur L. (1881). Note sur la maladie nouvelle provoquée par la salive d’un enfant mort de la rage. Bull. Acad. Natl. Méd..

[151] Rappuoli R. (2014). Inner Workings: 1885, the first rabies vaccination in humans. Proc. Natl. Acad. Sci. USA.

[152] Pasteur L. (1885). Méthode pour prévenir la rage après morsure. C. R. Acad. Sci..

[153] Pasteur L., Chamberland C.E., Roux E.P.P. (1884). Sur la rage. Bull. Acad. Natl. Méd..

[154] Suzor J.-R. (1888). Exposé pratique du traitement de la rage par la méthode Pasteur: historique et description de la rage, collection complète des communications de M. Pasteur, technique de sa méthode, resultats statistiques, etc..

[155] Plotkin S. (2011). History of Vaccine Development.

[156] Joseph Meister (1876–1940).

[157] Rupprecht C.E., Plotkin S.A. (2013). 29—Rabies vaccines. Vaccines.

[158] Dubail A. (1985). Joseph Meister, le premier être humain sauvé de la rage. Annu. Société Hist. Val Villé.

[159] Wolpert L. Experiments in Deceit. New York Times.

[160] Geison G.L. (2014). The Private Science of Louis Pasteur.

[161] Académie de Versailles Louis Pasteur: Quelques Textes. http://www.histoire.ac-versailles.fr/old/pedagogie/pasteur/pasteur_ressources.htm.

[162] Brunet J.-P. (2012). La rage envers Pasteur—Ou le révisionnisme en Sciences médicales. Bull. Assoc. Anc. Elèves Inst. Pasteur.

[163] Hansen B. (1998). America’s first medical breakthrough: How popular excitement about a French rabies cure in 1885 raised new expectations for medical progress. Am. Hist. Rev..

[164] Bazin H. (2011). Rabies or hydrophobia vaccine. Vaccination: A History.

[165] Schwartz M. (2008). Histoire et actualité du réseau international des Instituts Pasteur. Ann. Mines Responsab. Environ..

[166] Ilya Mechnikov—Biographical. http://www.nobelprize.org/nobel_prizes/medicine/laureates/1908/mechnikov-bio.html.

[167] Botvinkin A., Kosenko M., King A.A., Fooks A.R., Aubert M., Wandeler A.I. (2004). Chapter 5: Rabies in the European parts of Russia, Belarus and Ukraine.

[168] Ulyankina T., Cazenave P.-A., Talwar G. (1991). The Pasteur Institute and the advent of immunology in Russia (1880–1917). Immunology—Pasteur’s Heritage.

[169] Marie A.A. (1909). L’étude Experimentale de la Rage.

[170] Bernard P.-N. (1922). Les Instituts Pasteur d’Indochine.

[171] Dedet J.-P. (2001). Les Instituts Pasteurs D’outre-Mer. Cent Vingt Ansde Microbiologie Français.

[172] Guénel A. (1999). The creation of the first overseas Pasteur Institute, or the beginning of Albert Calmette’s Pastorian career. Med. Hist..

[173] Calmette A. (1891). Notes sur la rage en Indo-Chine et sur les vaccinations antirabiques pratiquées à Saïgon du 15 Avril au 1er Août 1891. Ann. Inst. Pasteur.

[174] Sun B.Z. (2014). Medicine as Colonial Enterprise: The Founding of the Pasteur Institute in Saigon, 1891.

[175] Smith T.G., Wu X., Franka R., Rupprecht C.E. (2011). Design of future rabies biologics and antiviral drugs. Adv. Virus Res..

[176] Cabot F. (1899). Report of experimental work on the dilution method of immunization from rabies. J. Exp. Med..

[177] Semple D. (1911). The Preparation of a Safe and Efficient Antirabic Vaccine.

[178] Chakrabarti P. (2010). “Living versus dead”: The Pasteurian paradigm and imperial vaccine research. Bull. Hist. Med..

[179] Wu X., Smith T.G., Rupprecht C.E. (2011). From brain passage to cell adaptation: The road of human rabies vaccine development. Expert Rev. Vaccines.

[180] Babes V., Lepp V. (1889). Recherches sur la vaccination antirabique.

[181] Hosty T.S., Kissling R.E., Schaeffer M., Wallace G.A., Dibble E.H. (1959). Human antirabies gamma globulin. Bull. World Health Organ..

[182] Habel K. (1957). Rabies prophylaxis in man. Public Health Rep..

[183] Habel K. (1945). Seroprophylaxis in experimental rabies. Public Health Rep..

[184] Anderson D.A. (2007). WHO guidelines dealing with immunoglobulin use impede rabies prevention. Asian Biomed..

[185] Expert Committee on Rabies (1957). Third Session.

[186] Koprowski H., Cox H.R. (1951). Recent developments in the prophylaxis of rabies. Am. J. Public Health Nations Health.

[187] Nagarajan T., Rupprecht C.E., Dessain S.K., Rangarajan P.N., Thiagarajan D., Srinivasan V.A. (2008). Human monoclonal antibody and vaccine approaches to prevent human rabies. Curr. Top. Microbiol. Immunol..

[188] Hanlon C.A., Niezgoda M., Morrill P.A., Rupprecht C.E. (2001). The incurable wound revisited: Progress in human rabies prevention?. Vaccine.

[189] Gogtay N., Thatte U., Kshirsagar N., Leav B., Molrine D., Cheslock P., Kapre S.V., Kulkarni P.S., SII RMab Author Group (2012). Safety and pharmacokinetics of a human monoclonal antibody to rabies virus: A randomized, dose-escalation phase 1 study in adults. Vaccine.

[190] Muhamuda K., Madhusudana S.N., Ravi V. (2007). Use of neutralizing murine monoclonal antibodies to rabies glycoprotein in passive immunotherapy against rabies. Hum. Vaccin..

[191] De Kruif J., Bakker A.B.H., Marissen W.E., Kramer R.A., Throsby M., Rupprecht C.E., Goudsmit J. (2007). A human monoclonal antibody cocktail as a novel component of rabies postexposure prophylaxis. Annu. Rev. Med..

[192] Van Dolleweerd C.J., Teh A.Y.-H., Banyard A.C., Both L., Lotter-Stark H.C.T., Tsekoa T., Phahladira B., Shumba W., Chakauya E., Sabeta C.T. (2014). Engineering, expression in transgenic plants and characterisation of E559, a rabies virus-neutralising monoclonal antibody. J. Infect. Dis..

[193] Both L., van Dolleweerd C., Wright E., Banyard A.C., Bulmer-Thomas B., Selden D., Altmann F., Fooks A.R., Ma J.K.-C. (2013). Production, characterization, and antigen specificity of recombinant 62-71-3, a candidate monoclonal antibody for rabies prophylaxis in humans. FASEB J. Off. Publ. Fed. Am. Soc. Exp. Biol..

[194] Müller T., Dietzschold B., Ertl H., Fooks A.R., Freuling C., Fehlner-Gardiner C., Kliemt J., Meslin F.X., Rupprecht C.E., Tordo N. (2009). Development of a mouse monoclonal antibody cocktail for post-exposure rabies prophylaxis in humans. PLoS Negl. Trop. Dis..

[195] (1935). Antirabies Treatment. Am. J. Public Health Nations Health.

[196] Levaditi C. (1913). Virus rabique et culture des cellules “in vitro”. C. R. Soc. Biol..

[197] Webster L.T., Clow A.D. (1937). Propagation of rabies virus in tissue culture. J. Exp. Med..

[198] Sokol F., Kuwert E., Wiktor T.J., Hummeler K., Koprowski H. (1968). Purification of rabies virus grown in tissue culture. J. Virol..

[199] Habel K. (1966). Laboratory techniques in rabies. Habel test for potency. Monogr. Ser. World Health Organ..

[200] Kaplan M.M., Koprowski H. (1973). Laboratory Techniques in Rabies.

[201] Fuenzalida E., Palacios R., Borgono J.M. (1964). Antirabies antibody response in man to vaccine made from infected suckling-mouse brains. Bull. World Health Organ..

[202] Molner J.G., Willson R.F., Kalish S. (1955). Rabies control in Detroit. Am. J. Public Health Nations Health.

[203] Schnurrenberger P.R., Anderson G.R., Russell J.H. (1967). Rapidity and magnitude of antibody response to duck-embryo rabies vaccine administered as a pre-exposure regimen. Bull. World Health Organ..

[204] Kaur M., Garg R., Singh S., Bhatnagar R. (2015). Rabies vaccines: Where do we stand, where are we heading?. Expert Rev. Vaccines.

[205] Wiktor T.J., Sokol F., Kuwert E., Koprowski H. (1969). Immunogenicity of concentrated and purified rabies vaccine of tissue culture origin. Proc. Soc. Exp. Biol. Med. Soc. Exp. Biol. Med. N.Y. N..

[206] Sikes R.K., Cleary W.F., Koprowski H., Wiktor T.J., Kaplan M.M. (1971). Effective protection of monkeys against death from street virus by post-exposure administration of tissue-culture rabies vaccine. Bull. World Health Organ..

[207] (2009). Expert Consultation on Rabies Post-Exposure Prophylaxis.

[208] Baer G.M., Abelseth M.K., Debbie J.G. (1971). Oral vaccination of foxes against rabies. Am. J. Epidemiol..

[209] Wells C.W. (1954). The control of rabies in Malaya through compulsory mass vaccination of dogs. Bull. World Health Organ..

[210] Kristensson K., Dastur D.K., Manghani D.K., Tsiang H., Bentivoglio M. (1996). Rabies: Interactions between neurons and viruses. A review of the history of Negri inclusion bodies. Neuropathol. Appl. Neurobiol..

[211] Negri Luzzani L. (1913). Le diagnostic de la Rage par la Démonstration du Parasite Spécifique—Résultat de Dix Ans d’Expérience (Première Partie). Ann. Inst. Pasteur J. Microbiol. Publiées Sous Patronage M Pasteur Par E Duclaux.

[212] Negri Luzzani L. (1913). Le diagnostic de la Rage par la Démonstration du Parasite Spécifique—Résultat de Dix Ans d’Expérience (Deuxième Partie). Ann. Inst. Pasteur J. Microbiol. Publiées Sous Patronage M Pasteur Par E Duclaux.

[213] Almeida J.D., Howatson A.F., Pinteric L., Fenje P. (1962). Electron microscope observations on rabies virus by negative staining. Virology.

[214] Matsumoto S. (1963). Electron microscope studies of rabies virus in mouse brain. J. Cell Biol..

[215] Matsumoto S. (1962). Electron microscopy of nerve cells infected with street rabies virus. Virology.

[216] Flamand A., Delagneau J.F. (1978). Transcriptional mapping of rabies virus in vivo. J. Virol..

[217] Tordo N., Poch O., Ermine A., Keith G., Rougeon F. (1988). Completion of the rabies virus genome sequence determination: Highly conserved domains among the L (polymerase) proteins of unsegmented negative-strand RNA viruses. Virology.

[218] Meslin F.-X., Kaplan M.M., Koprowski H., World Health Organization (1996). Laboratory Techniques in Rabies.

[219] Duong V., Tarantola A., Ong S., Mey C., Bourhy H., Dussart P., Buchy P. (2016). Laboratory diagnostics in dog-mediated rabies—An overview of performance and a proposed strategy in various settings. Int. J. Infect. Dis..

[220] Briggs D.J., Banzhoff A., Nicolay U., Sirikwin S., Dumavibhat B., Tongswas S., Wasi C. (2000). Antibody response of patients after postexposure rabies vaccination with small intradermal doses of purified chick embryo cell vaccine or purified Vero cell rabies vaccine. Bull. World Health Organ..

[221] Jaiiaroensup W., Lang J., Thipkong P., Wimalaratne O., Samranwataya P., Saikasem A., Chareonwai S., Yenmuang W., Prakongsri S., Sitprija V. (1998). Safety and efficacy of purified Vero cell rabies vaccine given intramuscularly and intradermally. (Results of a prospective randomized trial). Vaccine.

[222] Quiambao B.P., Dimaano E.M., Ambas C., Davis R., Banzhoff A., Malerczyk C. (2005). Reducing the cost of post-exposure rabies prophylaxis: Efficacy of 0.1 mL PCEC rabies vaccine administered intradermally using the Thai Red Cross post-exposure regimen in patients severely exposed to laboratory-confirmed rabid animals. Vaccine.

[223] Fekadu M., Shaddock J.H., Baer G.M. (1981). Intermittent excretion of rabies virus in the saliva of a dog two and six months after it had recovered from experimental rabies. Am. J. Trop. Med. Hyg..

[224] Starr L.E., Sellers T.F., Sunkes E.J. (1952). Apparent recovery of a dog from rabies. J. Am. Vet. Med. Assoc..

[225] Mshelbwala P.P., Ogunkoya A.B., Maikai B.V. (2013). Detection of rabies antigen in the saliva and brains of apparently healthy dogs slaughtered for human consumption and its public health implications in abia state, Nigeria. ISRN Vet. Sci..

[226] Cleaveland S., Barrat J., Barrat M.J., Selve M., Kaare M., Esterhuysen J. (1999). A rabies serosurvey of domestic dogs in rural Tanzania: Results of a rapid fluorescent focus inhibition test (RFFIT) and a liquid-phase blocking ELISA used in parallel. Epidemiol. Infect..

[227] Follmann E.H., Ritter D.G., Beller M. (1994). Survey of fox trappers in northern Alaska for rabies antibody. Epidemiol. Infect..

[228] Gilbert A.T., Petersen B.W., Recuenco S., Niezgoda M., Gómez J., Laguna-Torres V.A., Rupprecht C. (2012). Evidence of rabies virus exposure among humans in the Peruvian Amazon. Am. J. Trop. Med. Hyg..

[229] Weyer J., Msimang-Dermaux V., Paweska J.T., le Roux K., Govender P., Coertse J., Markotter W., Nel L.H., Blumberg L.H. (2016). A case of human survival of rabies, South Africa. South. Afr. J. Infect. Dis..

[230] Hattwick M.A., Weis T.T., Stechschulte C.J., Baer G.M., Gregg M.B. (1972). Recovery from rabies. A case report. Ann. Intern. Med..

[231] Willoughby R.E., Tieves K.S., Hoffman G.M., Ghanayem N.S., Amlie-Lefond C.M., Schwabe M.J., Chusid M.J., Rupprecht C.E. (2005). Survival after treatment of rabies with induction of coma. N. Engl. J. Med..

[232] Madhusudana S.N., Nagaraj D., Uday M., Ratnavalli E., Kumar M.V. (2002). Partial recovery from rabies in a six-year-old girl. Int. J. Infect. Dis. IJID Off. Publ. Int. Soc. Infect. Dis..

[233] (2010). Presumptive abortive human rabies—Texas, 2009. MMWR Morb. Mortal. Wkly. Rep..

[234] Porras C., Barboza J.J., Fuenzalida E., Adaros H.L., Oviedo A.M., Furst J. (1976). Recovery from rabies in man. Ann. Intern. Med..

[235] (2004). Recovery of a patient from clinical rabies—Wisconsin, 2004. MMWR Morb. Mortal. Wkly. Rep..

[236] (2012). Recovery of a patient from clinical rabies—California, 2011. MMWR Morb. Mortal. Wkly. Rep..

[237] Jackson A.C., Warrell M.J., Rupprecht C.E., Ertl H.C. J., Dietzschold B., O’Reilly M., Leach R.P., Fu Z.F., Wunner W.H., Bleck T.P. (2003). Management of rabies in humans. Clin. Infect. Dis. Off. Publ. Infect. Dis. Soc. Am..

[238] Jackson A.C. (2005). Recovery from rabies. N. Engl. J. Med..

[239] Wilde H., Hemachudha T., Jackson A.C. (2008). Viewpoint: Management of human rabies. Trans. R. Soc. Trop. Med. Hyg..

[240] Deressa A., Hussen K., Abebe D., Gera D. (2010). Evaluation of the Efficacy of Crude Extracts of *Salix subserrata* and *Silene macroselen* for the treatment of rabies in Ethiopia. Ethiop. Vet. J..

[241] Yamada K., Noguchi K., Komeno T., Furuta Y., Nishizono A. (2016). Efficacy of favipiravir (T-705) in rabies postexposure prophylaxis. J. Infect. Dis..

[242] Taylor L.H., Hampson K., Fahrion A., Abela-Ridder B., Nel L.H. (2017). Difficulties in estimating the human burden of canine rabies. Acta Trop..

[243] World Health Organization (1999). Recommended Standards and Strategies for Surveillance, Prevention and Control of Communicable Diseases. A82: Rabies.

[244] Hampson K., Coudeville L., Lembo T., Sambo M., Kieffer A., Attlan M., Barrat J., Blanton J.D., Briggs D.J., Cleaveland S. (2015). On behalf of the global alliance for rabies control partners for rabies prevention estimating the global burden of endemic canine rabies. PLoS Negl. Trop. Dis..

[245] World Health Organization (2016). Ebola Situation Report-30 March 2016.

[246] Abela-Ridder B., Knopf L., Martin S., Taylor L., Torres G., De Balogh K. (2016). 2016: The beginning of the end of rabies?. Lancet Glob. Health.

[247] Tarantola A., Ly S., In S., Ong S., Peng Y., Heng N.Y., Buchy P. (2015). Rabies vaccine and rabies immunoglobulin in Cambodia: Use and obstacles to use. J. Travel. Med..

[248] Tarantola A., Blanchi S., Cappelle J., Ly S., Chan M., In S., Peng Y., Hing C., Taing C.N., Ly S., Bourhy H., Buchy P., Dussart P., Mary J.-Y. (2017). Rabies postexposure prophylaxis (PEP) noncompletion after dog bites: estimating the unseen to meet the needs of the underserved. Am. J. Epidemiol.

